# Nano-sized Adsorbate Structure Formation in Anisotropic Multilayer System

**DOI:** 10.1186/s11671-017-2096-7

**Published:** 2017-05-05

**Authors:** Vasyl O. Kharchenko, Dmitrii O. Kharchenko, Vladimir V. Yanovsky

**Affiliations:** 1 Institute of Applied Physics, National Academy of Sciences of Ukraine, 58 Petropavlivska St., Sumy, Ukraine; 2 Institute for Single Crystals, National Academy of Sciences of Ukraine, 60 Nauky Avenue, Kharkiv, Ukraine

**Keywords:** Plasma-condensate system, Nanostructured thin films, Pattern formation, Nanostructures

## Abstract

In this article, we study dynamics of adsorbate island formation in a model plasma-condensate system numerically. We derive the generalized reaction-diffusion model for adsorptive multilayer system by taking into account anisotropy in transfer of adatoms between neighbor layers induced by electric field. It will be found that with an increase in the electric field strength, a structural transformation from nano-holes inside adsorbate matrix toward separated nano-sized adsorbate islands on a substrate is realized. Dynamics of adsorbate island sizes and corresponding distributions are analyzed in detail. This study provides an insight into details of self-organization of adatoms into nano-sized adsorbate islands in anisotropic multilayer plasma-condensate systems.

## Background

Nanostructured thin films with thickness ranging from nanometer to several micrometers are highly exploited in modern research areas, particularly to manufacture magnetoresistive sensors, memory devices, quantum dot lasers, and detectors which can be exploited at quantum communications. By using different techniques for fabrication of nanostructured thin films, one can get surface patterns of different morphology, such as nano-holes in adsorbate matrix [1] or separated adsorbate islands on a substrate [2]. Nowadays, the major exploitation of nanostructured thin films in the areas of energy generation, photovoltaics, photocatalysis, and sensor technology has tremendously been increasing.

Nanostructured thin films can be fabricated on different substrates by using different methods. Among them, one can issue ion-beam sputtering [3–6], when the thickness of the eroding thin film decreases; molecular beam epitaxy [7–12], and ion-beam or vapor deposition [13–20] when adsorbate forms a growing surface. For the last case, it was shown that the morphology of surface patterns, their statistical properties, and characteristic size can be controlled mainly by deposition flux and interaction strength between adatoms. Numerical studies of multilayer adsorptive system were reported mostly by considering isotropic and anisotropic vertical diffusion for gas-condensate system, assuming preferential motion of adatoms toward lower layers by minimizing the surface energy (see, for example, Ref. [16]). Anisotropic horizontal diffusion was studied by considering effects of horizontal electromigration, Refs. [21–23]. At the same time, there is no complete theoretical and numerical study of adsorbate islands growth in multilayer adsorptive systems with vertical anisotropic diffusion, when adatoms preferentially move toward upper layers. Such type of anisotropy is realized in plasma-condensate systems, which are widely used to fabricate small-sized separated adsorbate islands on a substrate [24, 25]. Here, the deposited atoms are ionized and the growing surface serves as an effective cathode. Hence, under the influence of electric field near the substrate, the main part of deposited atoms are re-evaporated to be later ionized again and returned back onto the adsorbate surface [26]. Such technological conditions lead to condensation of deposited atoms on the upper layers of the growing surface. It results to effective anisotropic diffusion of adatoms from the lower layers to the upper ones, caused by electric field presence. Processes of nanostructured thin film formation in plasma-condensate systems are mainly studied experimentally. At the same time, there is no detailed theoretical description of an influence of the electric field induced anisotropic diffusion of adsorbate onto dynamics and statistical properties of adsorbate islands.

In this article, we study dynamics of nano-sized adsorbate island formation in plasma-condensate systems and discuss an influence of electric field strength onto surface pattern morphology change by using numerical simulations. We will analyze statistical properties of adsorbate islands. It will be shown that an increase in the electric field strength leads to (i) structural transformation of the growing surface from nano-holes in adsorbate matrix toward separated adsorbate islands on a substrate, (ii) modality change in the distribution of adsorbate islands sizes, and (iii) decrease in the mean linear size of adsorbate islands.

## Methods

The generalized model for the adsorptive multilayer system can be written in the following form [16]:

1 $$ \partial_{t}\vec x(\mathbf{r},t)=\vec f(\vec x(\mathbf{r},t))-\nabla\cdot\vec{\mathbf{J}}(\vec x(\mathbf{r},t),\nabla).   $$

Here, $\vec x(\mathbf {r},t)=\{x_{i}(\mathbf {r},t)\}_{i=1}^{n}$ is the vector of the local adatom coverage on each *i*th layer of *n* layer system, *x*_*i*_(**r**,*t*)∈[0,1]; *t* is the time variable; **r**={*x*,*y*} is the space coordinate on each layer. The reaction term $\vec f(\vec x(\mathbf {r},t))=\{f_{i}(\vec x(\mathbf {r},t))\}_{i=1}^{n}$ describes adsorption, desorption, and adatom transfer between two neighbor layers on *i*th layer. Adsorbate flow vector $\vec {\mathbf {J}}(\vec x(\mathbf {r},t),\nabla)=\{\mathbf {J}_{i}(\vec x(\mathbf {r},t),\nabla)\}_{i=1}^{n}$ is responsible for diffusion and interaction of adsorbate.

Adsorption on *i*th layer is possible if one has (i) free cites on *i*th layer, (ii) non-zero coverage on (*i*−1)th layer, (iii) free sites on (*i*+*j*)th layers with *j*=1…*n*−*i*; adsorption rate *k*_*a*_ *p* is proportional to the gaseous phase pressure *p*. Adatoms can desorb from *i*th layer if there are (i) adatoms on *i*th layer; (ii) free cites on (*i*+*j*)th layers with *j*=1…*n*−*i*. Desorption rate on *i*th layer $k_{di}=k_{d}^{0}\exp (U_{i}(\mathbf {r})/T)$ is defined by desorption rate for noninteracting particles $k_{d}^{0}$ and interaction potential *U*_*i*_(**r**); we admit that only substratum-mediated interactions are possible. A transfer of adatoms from *i*th layer toward neighboring layers is possible only on free sites on (*i*−1)th and (*i*+1)th layers.

The total lateral flow of adsorbate on *i*th layer is:

2 $$ \mathbf{J}_{i\bot}=-D_{\bot}\nabla_{\bot} x_{i}-(D_{\bot}/T)x_{i}(1-x_{i})\nabla_{\bot} U_{i}.  $$

It takes into account free diffusion of adatoms (first term) and interaction part (second term), defined through the potential *U*_*i*_. This flow is possible only on the (1−*x*_*i*_) free sites; *D*_⊥_ is the constant for lateral diffusion, ∇_⊥_≡*∂*_*x*_+*∂*_*y*_. To define the interaction potential *U*_*i*_ of adsorbate, we exploit the receipt of self-consistent approximation used well in studying nano-sized island formation at condensation from gaseous phase [13–16, 27–37], pyramidal islands growth at molecular beam epitaxy [7–10], and formation of nano-sized clusters of point defects in solids [38–42].

In the framework of the self-consistent approximation, the interaction potential between adatoms separated by a distance *r* on the first layer counted from the substrate, reads $U_{1}=-\int u_{1}(r-r')x_{1}(r')\mathrm {d}r'$, where the integration is provided over the whole surface. The binary attraction potential *u*_1_(*r*) is assumed in symmetrical form, i.e., $\int r^{2n+1}u_{1}(r)\mathrm {d}r=0$. Following Ref. [31] for *u*_1_(*r*), we exploit Gaussian profile in the standard form:

3 $$ u_{1}(r)=\frac{2\epsilon_{1}}{\sqrt{4\pi r_{01}^{2}}}\exp\left(-\frac{r^{2}}{4r_{01}^{2}}\right).  $$

Here, *ε*_1_ is the interaction strength of adsorbate and *r*_01_ is the interaction radius. Next, by assuming that the diffusion length $L_{Di}=\sqrt {D_{i\bot }/k_{di}^{0}}$ is larger comparing to the interaction radius *r*_0*i*_^1^, and by taking into account that the coverage *x*_1_ behaves slowly comparing to *u*_1_(*r*) on interaction radius *r*_01_, we will use an expansion $\int u_{1}(\mathbf {r}-\mathbf {r}')x_{1}(\mathbf {r}')\mathrm {d}\mathbf {r}'\simeq \int u_{1}(\mathbf {r}-\mathbf {r}')\sum _{n} \frac {(\mathbf {r}-\mathbf {r}')^{n}}{n!}\nabla ^{n} x_{1}(\mathbf {r})\mathrm {d}\mathbf {r}'$. In this series, we retain only three non-vanishing terms:

4 $$\begin{array}{*{20}l} &\int u_{1}(r-r')x_{1}(r')\mathrm{d}r'\\ \simeq &\int u_{1}(r-r')\left[x_{1}(r)+\frac{(r-r')^{2}}{2!}\nabla^{2}x_{1}(r)\right.\\ &\left.\qquad+\frac{(r-r')^{4}}{4!}\nabla^{4}x_{1}({r})\right]{\mathrm d}{r}'. \end{array} $$

By substituting Eq.(3) into Eq.(4), we get:

5 $$\begin{array}{*{20}l} &\int u_{1}(r) x_{1}(r)\mathrm{d}r=2\epsilon_{1} x_{1},\\ \frac{1}{2} &\int u_{1}(r) r^{2}\nabla^{2} x_{1}(r)\mathrm{d}r=2\epsilon_{1} r_{01}^{2}\nabla^{2}x_{1},\\ \frac{1}{4!}&\int u_{1}(r) r^{4}\nabla^{4} x_{1}(r)\mathrm{d}r=\epsilon_{1} r_{01}^{4}\nabla^{4} x_{1}. \end{array} $$

By combining all terms, finally one has:

6 $$ \int u_{1}(\mathbf{r}-\mathbf{r}')x_{1}(\mathbf{r}'){\mathrm d}\mathbf{r}'\simeq\epsilon_{1} x_{1}(\mathbf{r})+\epsilon_{1}(1+r_{01}^{2}\nabla^{2})^{2}x_{1}(\mathbf{r}).  $$

Next, due to adsorbed particles (adatoms) are equivalent, we consider the case when the potential *u*(*r*) among particles in each layer is the same, i.e., *u*(*r*)≡*u*_*i*_(*r*) and *ε*≡*ε*_*i*_. This implies that *r*_0_≡*r*_0*i*_. Hence, in the framework of self-consistent approximation, the substratum-mediated interaction potential *U*_*i*_ on *i*th layer has the form [16]:

7 $$ U_{i}(\mathbf{r})\simeq-\epsilon x_{i-1}(\mathbf{r})\left\{ x_{i}(\mathbf{r})+(1+r_{0}^{2}\nabla_{\bot}^{2})^{2}x_{i}(\mathbf{r})\right\}  $$

with $\nabla _{\bot } U_{i}=-\epsilon x_{i-1}(\mathbf {r})\nabla _{\bot }\left \{ x_{i}(\mathbf {r})+(1+r_{0}^{2}\nabla _{\bot }^{2})^{2}x_{i}(\mathbf {r})\right \}$, *x*_0_=1. For multilayer systems, one should take into account vertical diffusion of adatoms between neighbor layers. This diffusion occurs only on free sites on the corresponding layers due to thermodynamic force influence. The corresponding diffusion is described by the term:

8) (9 $$\begin{array}{*{20}l} -\nabla\cdot \mathbf{J}_{i||}=D_{||}&\left[x_{i+1}(1-x_{i})-x_{i}(1-x_{i-1})\right.\\ &\left.+x_{i-1}(1-x_{i})-x_{i}(1-x_{i+1})\right], \end{array} $$

where *D*_||_ relates to diffusivity between layers.

By considering plasma-condensate system, one should take into account that the vertical diffusion of adatoms becomes anisotropic due to the applied electric field with the strength **E**=−∇*ϕ*, where *ϕ* is the electric potential difference. In such system, the substance is sputtered in the magnetron sputterer and accumulated near the substrate, which is located in a hollow cathode. Under the plasma influence, the main part of deposited atoms are re-evaporated to be later ionized again and returned onto an energetically favorable position on the upper adsorbate layer under the influence of electric field [26]. Therefore, such ionized atoms condense on the upper layers of growing surface. The corresponding additional vertical flux of adsorbate induced by electric field takes the form $\mathbf {J}_{i||}^{E}=\left (\mathbf {E}ZeD_{||}/T\right)x_{i}$, where *Z* is the valence of ion and *e* is the electron charge. By taking into account that this flux is possible only on free sites on neighbor layers, one has:

10 $$ -\nabla\cdot \mathbf{J}_{i||}^{E}=-uD_{||}\left[x_{i-1}(1-x_{i})-x_{i}(1-x_{i+1})\right],  $$

where *u*=|**E**|*Z* *e*/*T* denotes the anisotropy strength of vertical diffusion of adsorbate.

Next, due to all adatoms are equivalent (the same atoms), we assume *D*_||_=*D*_⊥_ and use the scaling parameters $t'\equiv tk_{d}^{0}$ and *r* ^′^=*r*/*L*_*D*_ and introduce dimensionless adsorption rate $\alpha \equiv k_{a}p/k_{d}^{0}$, interaction strength *ε*≡*ε*/*T*, and interaction radius *ρ*_0_=*r*_0_/*L*_*D*_. By taking into account all introduced terms, the dimensionless evolution equation for adsorbate concentration on *i*th layer has the form:

11 $$ \partial_{t}x_{i}=f_{i}(\vec x(\mathbf{r},t)) -\nabla\cdot\left[\mathbf{J}_{i\bot}+\mathbf{J}_{i||}+\mathbf{J}_{i||}^{E}\right]+\xi_{i}(\mathbf{r},t),  $$

where the corresponding reaction term on *i*th layer is $f_{i}=\alpha x_{i-1}\prod _{j=i}^{n}(1-x_{j})-x_{i}\prod _{j=i+1}^{n}(1-x_{j})e^{-2\varepsilon x_{i}x_{i-1}}$. For the boundary conditions for vertical diffusion, we admit that transitions *x*_1_⇔*x*_0_ and *x*_*n*_⇔*x*_*n*+1_ are forbidden. We include fluctuating terms *ξ*_*i*_ responsible for statistical description of the system dynamics 〈*ξ*_*i*_〉=0, 〈*ξ*_*i*_(**r**,*t*)*ξ*_*j*_(**r** ^′^,*t* ^′^)〉=*δ*_*ij*_ *δ*(*t*−*t* ^′^)*δ*(**r**−**r** ^′^).

We will focus our attention onto studying an influence of the anisotropy strength *u*, related to the electric field strength onto growth dynamics of anisotropic multilayer system and statistical properties of stationary adsorbate islands.

## Results and Discussion

In order to perform detailed study of processes of multi-layer adsorbate structure formation in a studied system, we make a discretization of Eq. (11) in three-dimensional space, where adsorbate condenses on the two-dimensional *L*×*L* substrate with *L*=512 cites. As was pointed out before, for metals and semiconductors, one has *L*_*D*_>*r*_0_. To perform accurate numerical modeling, following Refs. [15, 16, 30, 31], we use the relation *L*_*D*_/*r*_0_=40 with *r*_0_=0.5 that gives *L*=25.6*L*_*D*_. The time step satisfying the stability of the simulation algorithm is *Δ* *t*=10^−4^ in dimensionless units; the mesh size is *Δ* *l*=0.5. We use periodical boundary conditions for lateral diffusion. To study the dynamics of nano-sized pattern formation, we solve numerically the set of Eq. (11) on triangular lateral grid for each layer. To that end, the stochastic Heun algorithm has been implemented with double precision. As initial conditions, we take *x*_*i*_(**r**,0)=0 for *i*=2..*n*; 〈*x*_1_(**r**,0)〉=0, 〈(*δ* *x*_1_(**r**,0))^2^〉=0.1.

Previously for mono- and multi-layer adsorptive gas-condensate systems, it was shown that one can control the morphology of adsorbate structures by varying adsorption coefficient *α* and interaction strength *ε* [15, 16]. In this study, we are aimed to analyze an influence of anisotropy strength *u* onto statistical properties of adsorbate nano-islands. Hence, in all simulations, we fix *α*=0.2 and *ε*=4.

**Fig. 1:**
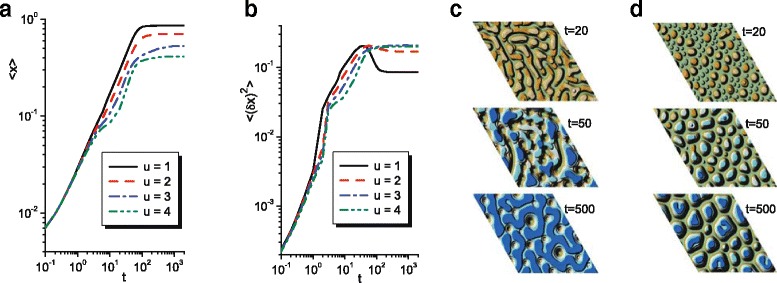
Results of numerical simulations of adsorbate self organization in plasma-condensate system. **a** Dynamics of the mean adsorbate concentration 〈*x*〉 at different values of anisotropy strength *u*. **b** Order parameter 〈(*δ* *x*)^2^〉 at different values of anisotropy strength *u*. Snapshots of the system evolution in **c** isotropic system at *u*=1 and **d** anisotropic system at *u*=3

Initially, let us consider dynamics of the mean adsorbate concentration over the whole system 〈*x*〉 and the related dispersion 〈(*δ* *x*)^2^〉≡〈*x* ^2^〉−〈*x*〉^2^. The last quantity plays a role of an order parameter for pattern formation (i) at 〈(*δ* *x*)^2^〉=0 adatoms homogeneously distributed on a substrate; (ii) if 〈(*δ* *x*)^2^〉 grows in time, then ordering processes accompanied by adsorbate island formation are realized; and (iii) at 〈(*δ* *x*)^2^〉=*c* *o* *n* *s* *t*, one has stationary patterns. Dynamics of the mean adsorbate concentration and related order parameter is shown in Fig. 1. It is seen that the mean concentration grows in time and attains the stationary value, which decreases with the anisotropy strength *u* growth (see Fig. 1 a). The order parameter grows in time that means formation of separated adsorbate clusters (see Fig. 1 b). In isotropic (*u*=1) and weakly anisotropic (*u*=2) systems, the quantity 〈(*δ* *x*)^2^〉 attains maximal value, decreases, and takes stationary value. A decreasing dynamics of an order parameter means a morphology change of patterns (re-arrangement of adsorbate islands). For the strong anisotropic system, 〈(*δ* *x*)^2^〉 increases monotonically to a stationary value (see curves for *u*=3 and *u*=4) which grows with anisotropy strength *u* increase. Typical snapshots of the isotropic (*u*=1) and anisotropic (*u*=3) systems evolution are shown in Fig. 1 c, d, respectively. Hereinafter, we present a quarter of lateral length of simulation grid. It follows that at *u*=1 adsorbate islands, formed at early stages, start to reorganize, forming adsorbate matrix with holes. At *u*=3 adsorbate, clusters grow in time forming well-defined separated islands. Hence, with an increase in anisotropy strength, one has structural transformation from the configuration of vacancy islands (holes) toward the configuration of adsorbate islands.

**Fig. 2:**
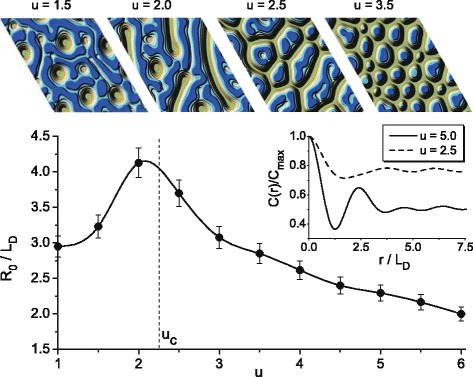
Dependence of the mean distance between structures *R*_0_ versus anisotropy strength. Snapshots in the stationary limit at different values of the anisotropy strength *u* are shown in the *top panel*. The corresponding stationary two-point correlation functions at different values of the anisotropy strength *u* are shown in insertion

To study such a morphological transformation in detail next, we provide statistical analysis considering stationary two-point correlation function *C*(*r*)=〈*x*(*r*)*x*(0)〉, which can be represented in a form: $C(r)=Ae^{-r/R_{c}}\cos (2\pi r/R_{0}+\phi)$, where *R*_*c*_ and *R*_0_ are the correlation radius and the mean distance between structures, respectively. The quantity *R*_0_ is useful to characterize the morphological transformation in a system. A dependence of the *R*_0_ in units of diffusion length *L*_*D*_ versus anisotropy strength *u* is shown in Fig. 2 with typical stationary snapshots for different *u*. It follows that *R*_0_ grows with an increase in the anisotropy strength. In the vicinity of *u*_*c*_, the mean distance between structures takes maximal value and decreases with further growth in *u*. Hence, at *u*=*u*_*c*_, one gets the picture like in phase separation with 〈*x*〉=1/2; at 1≤*u*<*u*_*c*_ during exposing, one gets holes in adsorbate matrix; and at *u*>*u*_*c*_, separated adsorbate islands will grow on a substrate. Typical stationary snapshots illustrating this morphological transformation are shown in the top in Fig. 2 at different values of *u*. Correlation functions *C*(*r*), manifesting oscillatory behavior with different period, are shown in insertion in Fig. 2. Similar behavior of the mean distance between structures *R*_0_ was observed with increase in the internal noise intensity in one-layer adsorptive gas-condensate model [14].

**Fig. 3:**
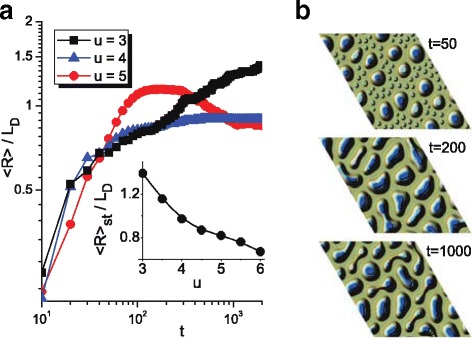
Evolution of the mean linear size of adsorbate islands 〈*R*〉 in units of diffusion length *L*_*D*_ on a half-height of the growing surface at different values of anisotropy strength *u* (**a**). Typical snapshots of the system evolution at *u*=5 and different time instances (**b**)

Next, we will discuss a change in the mean linear size of adsorbate islands 〈*R*〉 on a half-height of the growing surface with an increase in the anisotropy strength *u* and corresponding distributions of adsorbate islands over sizes *f*(*R*/〈*R*〉). Temporal dependencies 〈*R*(*t*)〉 in units of diffusion length *L*_*D*_ at different values of *u* are shown in Fig. 3 a. It is seen that at small values of anisotropy strength (see curve with black squares at *u*=3), the mean linear size 〈*R*〉 of adsorbate islands on a half-height of a growing surface grows in time even at large time scales, when the mean adsorbate concentration takes stationary value (see dash-dot curve for *u*=3 in Fig. 1 a). With an increase in the anisotropy strength *u* (see curve with blue triangles at *u*=4 in Fig. 3 a) after the stage of adsorbate island growth (increasing dynamics of 〈*R*〉), the mean linear size of adsorbate islands takes stationary value at large time scales. At large values of *u*, the dependence 〈*R*(*t*)〉 changes crucially (see curve with red circles at *u*=5 in Fig. 3 a): the mean linear size of adsorbate islands increases attaining its maximal value and then decreases to the stationary value. A decrease in 〈*R*(*t*)〉 means that the motion of adatoms to the upper layers at large *u* provides more compact structure of adsorbate islands. It follows that the stationary value of the mean linear size of adsorbate islands decreases with the anisotropy strength growth. The corresponding dependence 〈*R*_*st*_(*u*)〉 is shown in the insertion in Fig. 3 a. In Fig. 3 b, we show typical snapshots of the system evolution at *u*=5 and different time instants. It is seen that at a stage when the mean linear size grows in time, one can observe both large multilayer adsorbate islands and small islands on the first layer (see Fig. 3 b at *t*=50). During exposing, these small islands start to interact between themselves and with large ones. It results in rapid growth in the linear size of multilayer adsorbate island (see Fig. 3 b at *t*=200). At further exposing, strong anisotropy leads to a change in a shape of non-spherical adsorbate clusters. As a result, when the lateral thickness of the structure becomes less than the critical one, such structures start to divide (see Fig. 3 b at *t*=1000) that leads to decrease in the mean linear size of adsorbate islands 〈*R*〉.

**Fig. 4:**
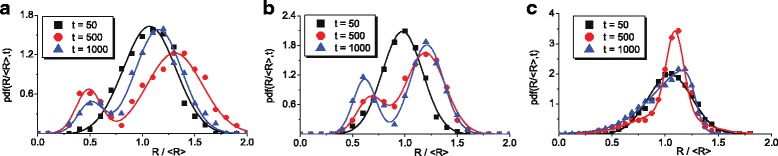
Evolution of adsorbate islands distribution function over re-normalized sizes on a half-height of a growing surface at different values of anisotropy strength: **a** *u*=3, **b** *u*=4, and **c** *u*=5

An evolution of distribution of adsorbate islands over sizes is shown in Fig. 4 a–c at *u*=3, *u*=4, and *u*=5, respectively. Initially, let us consider the case *u*=3. It follows that the unimodal Gaussian-like distribution centered at *R*=〈*R*〉 (black squares), realized at initial stages of the system evolution (*t*=50), transforms into bimodal one (red circles) at stage of adsorbate islands growth (*t*=500). Such unimodal-bimodal transformation of adsorbate island distribution means that during exposing, adsorbate islands are characterized by small and large sizes comparing to the mean value 〈*R*〉. At next, stages of the system evolution large islands continue to grow, whereas small ones can dissolve or they can be captured by larger islands leading to an increase in the mean size of adsorbate islands. These processes result in a decrease in a height of the peak located at *R*<〈*R*〉 (blue triangles) during exposing (see Fig. 4 a). At elevated values of the anisotropy strength *u*=4, the realized unimodal-bimodal transformation of a distribution function at initial stages becomes stable at exposing the height of the peak located at *R*<〈*R*〉 increases in time (see Fig. 4 b). It means that small adsorbate islands on a substrate remain stable and, as a result, the mean adsorbate island size attains the stationary value at large time scales (see curve at *u*=4 in Fig. 3 a). The corresponding bimodal distributions are well approximated by mixed Gaussian distribution represented as sum of two Gaussians. Similar bimodal island size distributions were found in one-layer vapor-condensate adsorptive model [15]. Experimentally, bimodal distributions were observed at adsorption and reaction of *R* *h*(*C* *O*)_2_ (acac) on *A* *l*_2_ *O*_3_/*N* *i*_3_ *A* *l* (111) [43] and at the formation of *Ag* nanoclusters supported on *T* *i* *O*_2_ (110) [44]. At large values of anisotropy strength, one has following transformations of the distribution function (see Fig. 4 c at *u*=5): initially, unimodal Gaussian distribution does not change its modality but becomes non-symmetrical. This distribution is characterized by the heavy tail at *R*<〈*R*〉 and has a form of well-known Lifshitz-Slyozov-Wagner distribution [45, 46].

**Fig. 5:**
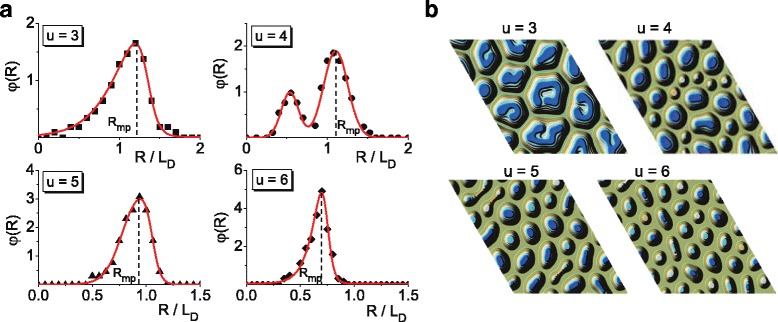
Distribution functions of adsorbate islands over sizes in stationary limit on a half-height of a growing surface **a** and typical snapshots of surface patterns **b** at different values of anisotropy strength *u*

Finally, we will discuss an influence of the anisotropy strength *u* onto stationary distribution *φ*(*R*) of adsorbate islands over sizes, measured in units of diffusion length *L*_*D*_. In Fig. 5 a we present calculated distributions *φ*(*R*) for different values of anisotropy strength *u*. It is seen that at small *u*, the distribution *φ*(*R*) has non-symmetrical form with respect to the most probable size *R*_*mp*_. Hence, bimodal distribution shown in Fig. 3 a becomes unimodal in large time limit with a form of Lifshitz-Slyozov-Wagner distribution. With an increase in *u* from 3 to 4, one has modality change of the distribution of adsorbate islands over sizes in stationary limit: distribution *φ*(*R*) is bimodal and is characterized by one peak located at small *R* and another one at large *R*. It means that in the stationary regime, one can observe on a substrate large amount of large adsorbate clusters and small number of small clusters. The most probable value of the linear size of adsorbate structures *R*_*mp*_, which correspond to the largest peak of *φ*(*R*) decreases, comparing to the case of *u*=3. With an increase in the anisotropy strength, the stationary distribution *φ*(*R*) transforms back to unimodal Lifshitz-Slyozov-Wagner distribution and the most probable size *R*_*mp*_ decreases (see plot at *u*=5). At large *u*, both the most probable value *R*_*mp*_ and the dispersion of this unimodal distribution decrease. Last means that most of adsorbate structures are characterize by the same linear size.

In Fig. 5 b, we present typical snapshots of surface patterns at different values for anisotropy strength *u*. It follows that at small anisotropy *u*=3, the surface is characterized by large adsorbate clusters. In the case of *u*=4, both large and small adsorbate clusters are realized. With a further increase in *u*, adsorbate clusters become smaller with preferentially spherical lateral shape; they tend to have equivalent sizes.

## Conclusions

In this paper, we have studied processes of adsorbate cluster formation in model plasma-condensate system by numerical simulations. We have studied the surface pattern morphology change by varying the anisotropic strength of vertical diffusion of adatoms related to the electric field strengths. An electric field influence was modelized by anisotropy in motion of adatoms between neighbor layers with preferential motion toward the upper layers.

It is found that with an increase in the anisotropic strength, the stationary adsorbate concentration on a substrate decreases and the growing surface becomes more ordered. By studying the correlation properties of a growing surface, it is shown that an increase in the anisotropic strength leads to the structural transformation of the growing surface morphology: from patterns characterized by holes inside adsorbate matrix toward separated adsorbate structures on a substrate. With an increase in the anisotropic strength, the mean distance between structures initially increases, indicating a decrease in the size of holes, and after reaching maximal value, which corresponds to the picture of spinodal decomposition, decreases.

It is shown that depending on the anisotropic strength, one gets different dynamics of adsorbate island growth. At initial stages of the system evolution, the mean size of adsorbate islands grows in time. Here, the distribution of adsorbate islands over sizes has Gaussian profile, centered around the mean size. At small values of the anisotropic strength, the mean linear size of adsorbate islands grows in time even at large time scales. During exposing, the Gaussian distribution changes the modality and in the stationary limit again becomes unimodal type of Lifshitz-Slyozov-Wagner distribution. At intermediate values of the anisotropic strength, the mean size of adsorbate islands attains stationary value at large times, when the growing stage is finished. The corresponding distribution transforms into bimodal one which is stable in the stationary limit. In such a case in the stationary limit, stable adsorbate islands are characterized by small and large sizes comparing to the mean size. At large values of the anisotropic strength, the Gaussian unimodal distribution of adsorbate islands over sizes transforms to the Lifshitz-Slyozov-Wagner distribution.

We have shown that with an increase in the anisotropic strength, both the mean size and the most probable size of adsorbate islands on a half-height of the growing surface decrease. The mean size of adsorbate islands varies from 0.75 up to 1.25 of diffusion length, which is from 10^−7^ *m* for metals and 10^−6^ *m* for semiconductors. Our obtained results for the linear size of adsorbate islands and distribution of adsorbate islands over sizes are in good quantitative correspondence with experimental studies for silicon and aluminum condensation in accumulative ion-plasma devices [24–26].

Obtained within this work results can be useful to predict self-organization of adatoms into 3D clusters in plasma-condensate systems for different materials (metals, semiconductors, etc.). We expect that our nontrivial findings will stimulate further theoretical and experimental studies of nano-size adsorbate island formation in anisotropic multilayer systems.

## Endnote

^1^ For metals and semiconductors, one has *r*_0_∼10^−9^÷10^−8^ m and *L*_*D*_∼10^−7^÷10^−6^ m.
